# In vitro inhibitory activities of selected Australian medicinal plant extracts against protein glycation, angiotensin converting enzyme (ACE) and digestive enzymes linked to type II diabetes

**DOI:** 10.1186/s12906-016-1421-5

**Published:** 2016-11-04

**Authors:** Permal Deo, Erandi Hewawasam, Aris Karakoulakis, David J. Claudie, Robert Nelson, Bradley S. Simpson, Nicholas M. Smith, Susan J. Semple

**Affiliations:** 1 School of Pharmacy and Medical Science, University of South Australia, Adelaide, South Australia 5000 Australia; 2 Chuulangun Aboriginal Corporation, PMB 30, Cairns Mail Centre, Cairns, Queensland 4870 Australia; 3 Flinders Centre for Innovation in Cancer, Flinders University, Bedford Park, South Australia, 5042 Australia; 4 Quality Use of Medicines and Pharmacy Research Centre, University of South Australia, GPO Box 2471, Adelaide, South Australia 5001 Australia; 5 Current address: Foodplus Research Centre, School of Agriculture, Food and Wine, University of Adelaide, South Australia, 5064 Australia

**Keywords:** Phenolics, Flavonoids, Antioxidant activities, Antiglycation, α-amylase, α-glucosidase, Angiotensin converting enzyme, Aboriginal, Traditional medicine, Local knowledge

## Abstract

**Background:**

There is a need to develop potential new therapies for the management of diabetes and hypertension. Australian medicinal plants collected from the Kuuku I’yu (Northern Kaanju) homelands, Cape York Peninsula, Queensland, Australia were investigated to determine their therapeutic potential. Extracts were tested for inhibition of protein glycation and key enzymes relevant to the management of hyperglycaemia and hypertension. The inhibitory activities were further correlated with the antioxidant activities.

**Methods:**

Extracts of five selected plant species were investigated: *Petalostigma pubescens*, *Petalostigma banksii*, *Memecylon pauciflorum*, *Millettia pinnata* and *Grewia mesomischa.* Enzyme inhibitory activity of the plant extracts was assessed against α-amylase, α-glucosidase and angiotensin converting enzyme (ACE). Antiglycation activity was determined using glucose-induced protein glycation models and formation of protein-bound fluorescent advanced glycation endproducts (AGEs). Antioxidant activity was determined by measuring the scavenging effect of plant extracts against 1, 1-diphenyl-2-picryl hydrazyl (DPPH) and using the ferric reducing anti-oxidant potential assay (FRAP). Total phenolic and flavonoid contents were also determined.

**Results:**

Extracts of the leaves of *Petalostigma banksii* and *P. pubescens* showed the strongest inhibition of α-amylase with IC_50_ values of 166.50 ± 5.50 μg/mL and 160.20 ± 27.92 μg/mL, respectively*.* The *P. pubescens* leaf extract was also the strongest inhibitor of α-glucosidase with an IC_50_ of 167.83 ± 23.82 μg/mL. Testing for the antiglycation potential of the extracts, measured as inhibition of formation of protein-bound fluorescent AGEs, showed that *P. banksii* root and fruit extracts had IC_50_ values of 34.49 ± 4.31 μg/mL and 47.72 ± 1.65 μg/mL, respectively, which were significantly lower (*p* < 0.05) than other extracts. The inhibitory effect on α-amylase, α-glucosidase and the antiglycation potential of the extracts did not correlate with the total phenolic, total flavonoid, FRAP or DPPH. For ACE inhibition, IC_50_ values ranged between 266.27 ± 6.91 to 695.17 ± 15.38 μg/mL.

**Conclusions:**

The tested Australian medicinal plant extracts inhibit glucose-induced fluorescent AGEs, α-amylase, α-glucosidase and ACE with extracts of *Petalostigma s*pecies showing the most promising activity. These medicinal plants could potentially be further developed as therapeutic agents in the treatment of hyperglycaemia and hypertension.

**Electronic supplementary material:**

The online version of this article (doi:10.1186/s12906-016-1421-5) contains supplementary material, which is available to authorized users.

## Background

Type II diabetes mellitus is a chronic disease characterised by high blood glucose level or hyperglycaemia. It is a major health concern around the world with co-morbidities such as obesity, hyperlipidemia, hypertension and cardiovascular diseases [1]. Currently, 422 million people worldwide are suffering from diabetes and this is expected to reach 642 million by 2040 [2, 3]. In Australia, 3.2 million people live with Type II diabetes, and it is known to be the 6th leading cause of death [2, 4]. For Australian Indigenous peoples, rates of diabetes and high blood sugar levels are between three and five times higher than those of non-Indigenous Australians [5].

Non-insulin dependent hyperglycaemia characterises the early stages of type II diabetes due to the increased breakdown of starch by α-amylase and absorption of glucose by α-glucosidase [6, 7]. The inhibition of these enzymes can significantly decrease the postprandial increase in blood glucose level after a mixed carbohydrate diet, hence it is an important strategy for the management of hyperglycaemia linked to type II diabetes [7]. Increasing evidence has shown that prolonged exposure to elevated glucose induces the production of free radicals, particularly reactive oxygen species (ROS) through glucose autoxidation and protein glycation [8, 9]. Glycation is a non-enzymatic reaction between free amino groups of proteins and carbonyl groups of reducing sugars, through the formation of Schiff bases. The unstable Schiff bases further rearrange to produce reversible Amadori products, promoting the formation of heterogeneous compounds collectively known as advanced glycation endproducts (AGEs) [10, 11]. Protein glycation plays significant roles in the initiation and progression of micro and macro-vascular complications associated with the pathogenesis of age-related diseases [10, 12]. The interactions of AGEs with receptors of AGEs (known as AGE-RAGE interactions) directly activate multiple intracellular signalling, gene expression and the secretion of pro-inflammatory molecules accompanied by increasing free radicals that contribute towards pathologic complications related to diabetes such as atherosclerosis, nephropathy, retinopathy and cataract [10–12]. One of the long-term complications of type II diabetes is arterial hypertension, which may eventually result in cerebrovascular accidents and cardiovascular diseases.

Angiotensin I-converting enzyme (ACE) plays an important part in the regulation of blood pressure and normal cardiovascular functions by two different reactions. It catalyses the conversion of angiotensin I to angiotensin II and inactivates the vasodilator bradykinin, which is conducive to lower blood pressure [13, 14]. Inhibition of ACE is considered to be a useful therapy for the control of blood pressure in hypertensive patients.

Various pharmacological approaches have been used in the management of diabetes via different modes of action such as: acarbose, miglitol, and voglibose which inhibit α-amylase and α-glucosidase activity [15, 16]; aminoguanidine for antiglycation in alleviating diabetic complications [17, 18]; and captopril which inhibits ACE for the treatment of hypertension [19]. However, none of the currently available inhibitors for clinical use are devoid of severe side effects [16, 18]. Therefore, alternative treatments including the use of traditional medicinal plants are now becoming an attractive approach for the treatment of postprandial hyperglycaemia and its related complications [20–23].

Australia is classified as one of the world’s 17 megadiverse countries, possessing rich plant biodiversity, with many species endemic to the continent [24]. Moreover, Aboriginal people across Australia hold significant customary medicinal knowledge associated with the use of different plant species for indications including infection and inflammatory conditions [25]. The Cape York Peninsula region in Far North Queensland is home to a diverse range of medicinal plant species. An ongoing collaborative research project exists between researchers at University of South Australia and Traditional Owners of the Northern Kaanju (Kuuku I’yu) homelands in Cape York Peninsula, Queensland. This research aims to improve social, health and economic outcomes for the Aboriginal people linked to these homelands through the development of medicinal plant products [26]. It also seeks to protect and restore biocultural diversity on homelands by bringing together local and traditional knowledge held by Aboriginal people with scientific knowledge [27]. Through investigation of the activities of medicinal plants, the project has potential to improve health outcomes for the broader community through the discovery of new therapeutic agents.

We have previously investigated several plant species for anti-inflammatory activities [25, 28] as Kuuku I’yu Traditional Owners have used these plants for treating diseases associated with inflammation [29]. Traditional medicinal plants previously shown to have anti-inflammatory properties have also been reported to exhibit blood-glucose-lowering and antiglycation effects [30–33]. In the present study, we investigated the effect of selected Australian medicinal plant extracts for α-amylase, α-glucosidase, and ACE inhibition and antiglycation related to hyperglycaemia. Correlations of the inhibitory activities with the total phenolic, total flavonoid and antioxidant activities were also examined.

## Methods

### Chemicals

Folin-Ciocalteu, gallic acid, ascorbic acids, quercetin, butyl hydroxyl toluene (BHT), 1,1-diphenyl-2-picrylhydrazyl (DPPH), bovine serum albumin (BSA), aminoguanidine, captopril, pancreatic α-amylase, yeast α-glucosidase enzyme, *p*-nitrophenyl-α-D-glucopyranoside, acarbose, furanacroloyl-Phe-Glu-Glu (FAPGG) and trichloroacetic acid (TCA) were purchased from Sigma-Aldrich (Sydney, Australia). All other chemicals used were of analytical or HPLC grade.

### Plant materials and extractions

Medicinal plants were collected on the Kuuku I’yu homelands. The Kuuku I’yu (Northern Kaanju) or Kaanichi Pama are “inland” people belonging to the highlands of central Cape York Peninsula, Northern Australia. Their “homelands” (also called *Ngaachi*) encompass some 840,000 hectares of diverse vegetation types. The land contains natural features that have national and international conservation significance. The Kuuku I’yu people are inexorably linked to the land and its resources which they value for their physical and spiritual well-being. Today, people continue to utilize these natural resources for their diet, cultural activities, and medicine and healing [27].

The plant species examined in this study were selected according to their medicinal uses as described as part of local knowledge by the Kuuku I’yu Northern Kaanju Traditional Owners (Table 1). *Grewia mesomischa* was formally identified by David Halford, Queensland Herbarium. Other plant species were formally identified by project ethnobotanist (author Dr Nicholas Smith) and voucher specimens were lodged and Western scientific names confirmed at Queensland Herbarium, Brisbane, Australia (Table 1).

**Table 1:** Different plant species and parts used in this study and local medicinal uses

Family	Plant species name	Part extracted	Uses according to local medicinal knowledge	Voucher number
Picrodendraceae	*Petalostigma banksii* Britten & S.Moore	Leaves, fruits, roots	Toothache, mouth inflammation	BRI AQ0737556 AQ0749696
	*Petalostigma pubescens* Domin	Leaves, fruits	Toothache, mouth inflammation	BRI AQ0737696
Melastomataceae	*Memecylon pauciflorum* Blume *var. pauciflorum*	Leaves	Skin infection and inflammation	BRI AQ0737545
Fabaceae	*Millettia pinnata* (L.) Panigrahi	Inner bark	Skin sores, inflammation and pruritis	BRI AQ0783017
Malvaceae	*Grewia mesomischa* Burret	Root bark	Stomach ache	BRI AQ0783018

Leaves, fruits, roots or bark were air dried in the shade immediately after collection, loosely packed in paper bags then transported to the laboratory for extraction. Extractions were performed as previously described [28]. Briefly, samples were extracted for 24 h using 80 % (v/v) ethanol. A solvent to plant material ratio of 10:1 was used for leaves and fruit and a ratio of 5:1 was used for roots/bark. After 24 h the first extract portion was removed and a second equivalent volume of fresh solvent was added to the plant material and allowed to extract for a further 24 h. The second portion was then decanted and the plant material was washed with 100 mL of solvent. The combined ethanolic extracts and wash were filtered using Whatman (#1) filter paper. The extract was concentrated using a rotary evaporator under reduced pressure below 40 °C, then freeze dried and stored at −20 °C.

### Phytochemical analysis

#### Total phenolic content

Total phenolic content in the extracted samples was determined as described elsewhere [7] with minor modifications. Briefly, sample extracts (0.5 mg/mL, 60 μL) and Folin-Ciocateu’s reagent (60 μL) were mixed and incubated at room temperature for 5 min. After incubation, 60 μL of Na_2_CO_3_ (10 % w/v) was added and the reaction mixture was further incubated for 60 min at room temperature in the dark. After incubation, the absorbance was measured at 760 nm. Gallic acid was used as standard and phenolic content expressed as μg gallic acid equivalents (GAE)/mg of sample weight.

#### Total flavonoid content

Flavonoid content in the extracted samples was estimated according to a previous method [7]. Briefly, sample extracts (0.5 mg/mL, 50 μL) were mixed with aluminium chloride solution (10 % (w/v), 20 μL), sodium acetate (1 M, 20 μL) followed by absolute ethanol (150 μL). After incubation for 30 min at room temperature in dark, the absorbance was measured immediately at 450 nm. Quercetin was used as standard and the results expressed as μg quercetin equivalents (QE)/mg of sample weight.

### In vitro antioxidant assays

#### Ferric reducing antioxidant potential (FRAP) assay

The ferric reducing activity of the extracts was estimated based on a modified FRAP procedure described previously [34]. Sample extracts (0.5 mg/mL, 20 μL) were mixed with 20 μL of 0.2 M potassium phosphate buffer (pH 7.2) and potassium ferricyanide (1 % w/v, 20 μL) followed by incubation at 50 °C for 20 min. After incubation, TCA (10 % w/v, 20 μL), distilled water (75 μL) and ferric chloride (0.1 % w/v, 20 μL) were added and the reaction mixture was further incubated for 30 min at room temperature in the dark. Absorbance was recorded at 630 nm. Ascorbic acid (0–250 μg/mL) was used to develop a standard curve and the results expressed as ascorbic acid equivalents per mg sample (μg AAE/mg).

#### 1,1-diphenyl-2-picrylhydrazyl (DPPH) radical inhibition

DPPH radical scavenging method was determined by an assay modified from da Silva Pinto et al. [35]. Sample extracts (0.5 mg/mL, 50 μL) and freshly prepared DPPH in methanol (0.2 mM, 150 μL) were mixed and incubated for 60 min at room temperature in the dark. After incubation, absorbance was recorded at 490 nm. BHT (0.5 mg/mL) was used as the positive control. DPPH activity was expressed as a percentage (%) inhibition of radical formation [35].

### In vitro antiglycation assay

Antiglycation activity was determined using glucose-induced protein glycation in vitro models. Aliquots (250 μL) of BSA (10 mg/mL, final concentration) in 0.2 M phosphate buffer (pH 7.4, containing 0.02 % sodium azide) were pre-incubated with sample extracts (50 μL, 0–500 μg/mL, final concentration) for 30 min at room temperature. After pre-incubation, 200 μL of glucose (100 mM final concentration) in 0.2 M phosphate buffer (pH 7.4, containing 0.02 % sodium azide) were added and the reaction mixture was incubated at 37 °C for 3 weeks. After incubation, protein was extracted by adding TCA (20 % w/v, 400 μL) to the glycated sample and the reaction mixture was kept at 4 °C for 10 min before centrifugation (1000 × g) for 10 min. The pellet was redissolved in alkaline phosphate buffer (0.2 M, pH 10, 2000 μL) and fluorescent AGEs were measured using a multimode plate reader (EnSpire, PerkinElmer, USA). The fluorescent intensity was measured at 370 nm (excitation) and 440 nm (emission). Aminoguanidine (30 mM, final concentration) was used as a positive control. The antiglycation was expressed as percentage inhibition of fluorescent AGE (%) = [(F_control_-F_sample_)/F_control_)] × 100.

### In vitro antidiabetic assays

#### Pancreatic α-amylase inhibition

The pancreatic α-amylase inhibition assay was modified from a previous study [21]. Different concentrations of sample extracts (125 μL) were mixed with 125 μL of α-amylase solution (0.5 mg/mL in 0.1 M sodium phosphate buffer, pH 6.9) and the reaction mixture was pre-incubated at 37 °C for 10 min. After pre-incubation, 25 μL of starch solution (1 % w/v) was added every 10 seconds to a total of 125 μL. The reaction mixture was further incubated at 37 °C for 20 min. After incubation the reaction was stopped by adding 250 μL of dinitrosalicylic reagent (1 % 3,5-dinitrosalicylic acid, 0.2 % phenol, 0.05 % Na_2_SO_3_ and 1 % NaOH in aqueous solution) to the reaction mixture. The reaction mixtures were heated at 100 °C for 10 min. Thereafter, 250 μL of potassium sodium tartarate solution (40 %) was added to the mixtures to stabilize the colour. After cooling to room temperature, absorbance of the reaction mixture was recorded at 540 nm. Acarbose (different concentrations) was used as positive control. Results were expressed as percentage (%) amylase inhibition = [(A_Control540_-A_Samples540nm)_/A_Control540nm_)] × 100.

#### α-glucosidase inhibition

The α-glucosidase inhibition assay was modified from a previous study [35]. Briefly, 50 μL of sample extracts (different concentrations) were mixed with 100 μL of yeast α-glucosidase enzyme (0.2 U/mL in 0.1 M potassium phosphate buffer solution, pH 6.9) and incubated at 37 °C for 30 min. After pre-incubation, 50 μL of *p*-nitrophenyl-α-D-glucopyranoside solution (5 mM) and the reaction mixture were further incubated at 37 °C for 30 min. Sodium carbonate solution (0.1 M, 60 μL) was added to the reaction mixture and incubated again at 37 °C for 20 min. Before and after incubation, absorbance readings were recorded at 405 nm. Acarbose (0.25–100 μg/mL) was used as a positive control. Results were expressed as described for α-amylase inhibition.

### Angiotensin converting enzyme (ACE) inhibition assay

The ACE inhibition assay was carried out using furanacroloyl-Phe-Glu-Glu (FAPGG) as described previously [13]. ACE inhibition was evaluated using different concentrations of sample extracts. The colour intensity was measured at 340 nm. Captopril was used as a positive control.

### Data analysis

All analysis was done in triplicates and each experiment was repeated three times. Results were expressed as mean ± standard deviation (SD). The IC_50_ was defined as the concentration of the samples causing 50 % inhibition of enzymes and protein glycation and was estimated by non-linear regression analysis using Graph Pad Prism software (San Diego, CA, USA, Version 6.03). Differences were evaluated by one-way analysis of variance (ANOVA) followed by Tukey’s multiple comparisons test, and *p* < 0.05 was considered to be significant. Linear regression analysis was determined with Pearson’s correlation coefficient (r^2^) to compare the relationship between antioxidants versus antiglycation properties; and antioxidant activities versus enzyme inhibition properties.

## Results and discussion

Hyperglycaemia is still considered the principal cause of diabetic complications attributed to increased production of ROS, breakdown of starch by α-amylase, absorption of glucose by α-glucosidase and the formation of AGEs, among others. Failure of existing antidiabetic drugs due to complications and side effects are forcing researchers to look for complementary medicines for management of diabetes. In recent years, plant-derived medicines worldwide have had a great deal of attention, due to their antioxidant properties, enzyme inhibitory activities, fewer side effects in comparison to conventional medicines and their economic viability [36].

In Australia, the investigation of Aboriginal traditional plant medicines for their potential to be developed into therapeutic products is gaining momentum. However, only a limited number of species have been extensively studied. There has been limited previous research on Australian Aboriginal medicinal plants for activity against enzymes relevant to carbohydrate metabolism [21, 37] and to our knowledge, no previous studies on Australian medicinal plants for antiglycation activities and inhibition of ACE. Our research is a collaboration between Aboriginal and University-based researchers with research into the efficacy and safety of their traditional plant medicines driven and guided by Traditional Owners. More broadly, our research aims to provide a framework for addressing issues that arise when considering the development of Australian medicinal plants into therapeutic products. This includes appropriate recognition of local and traditional knowledge and Indigenous Intellectual Property ownership and, should plant products be commercialised, the fair and equitable return of benefits back to the Traditional Owners [26].

In the current study, we investigated 80 % v/v aqueous ethanol extracts of five plant medicines, *Petalostigma banksii* (leaves, fruits and roots)*; Petalostigma pubescens* (leaves, fruits)*; Memecylon pauciflorum var. pauciflorum* (leaves)*; Millettia pinnata* (inner bark)*; and Grewia mesomischa* (root bark) for antiglycation and inhibitory potential against enzymes relevant to metabolic syndrome. The total phenolic flavonoid and the antioxidant activities of the extracts were also determined and correlated.

### Total phenolic, total flavonoid and antioxidant potential

We first examined the extracts to determine their content of phenolics and flavonoids. Phenolic and flavonoid compounds are secondary metabolites ubiquitously found in different parts of plants that commonly exhibit antioxidant activities. The total phenolic content of the tested plant extracts ranged from 72.36 ± 15.84 to 347.87 ± 14.90 μg GAE/mg (Table 2). Among the leaves, fruits and bark extracts, the highest total phenol content was in *P. banksii* fruits, whereas *M. pinnata* inner bark extracts had the lowest (Table 2). The extracts contained flavonoids in the range from 3.36 ± 0.87 to 26.30 ± 2.75 μg QE/mg (Table 2). Interestingly, *P. pubescens* fruits had the lowest flavonoid content, while the leaves of the same plant had the second highest flavonoid content among the selected medicinal plants*.*

**Table 2:** Total phenolic, flavonoid and FRAP contents in the selected Australian medicinal plant extracts

Plant name	Total phenolic (μg GAE/mg)	Flavonoid content (μg QE/mg)	FRAP (μg AAE/mg)
*Petalostigma banksii*			
Leaves	333.70 ± 11.95^e^	14.66 ± 3.25^b^	449.98 ± 41.74^d^
Fruits	347.87 ± 14.90^e^	7.02 ± 4.49^ab^	453.14 ± 38.19^d^
Roots	323.53 ± 12.16^e^	19.38 ± 5.38^bc^	235.68 ± 9.36^bc^
*Petalostigma pubescens*			
Leaves	276.96 ± 13.84^d^	22.64 ± 4.32^c^	453.30 ± 51.79^d^
Fruits	112.29 ± 5.34^b^	3.36 ± 0.87^a^	193.34 ± 7.01^b^
*Memecylon pauciflorum var. pauciflorum*			
Leaves	140.24 ± 5.28^b^	26.30 ± 2.75^c^	311.42 ± 23.16^c^
*Millettia pinnata*			
Inner bark	72.36 ± 15.84^a^	10.01 ± 4.05^ab^	26.46 ± 1.73^a^
*Grewia mesomischa*			
Root bark	200.71 ± 5.52^c^	10.61 ± 3.83^ab^	214.52 ± 21.79^b^

Values are expressed as mean ± SD, *n =* 3

*GAE* Gallic acid equivalence, *QE* Quercetin equivalence, *FRAP* ferric reducing antioxidant potential, *AAE* ascorbic acid equivalence

Data in the same column marked with different letters were significantly different (*p* < 0.05)

**Fig. 1:**
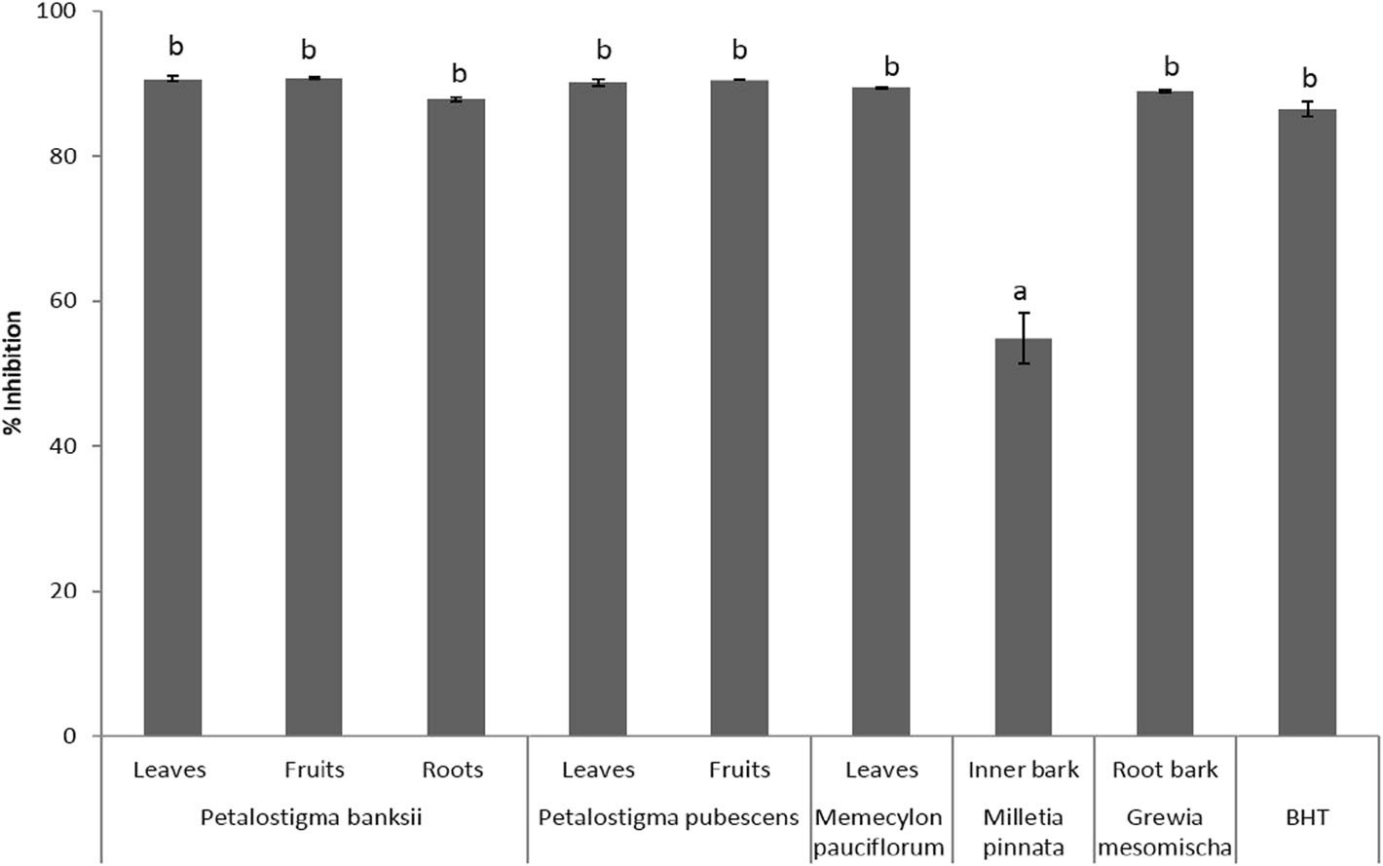
DPPH radical scavenging activity (%) of selected Australian medicinal plant extracts tested at 0.5 mg/mL. Values are expressed as means ± SD, *n =* 3. Data marked with different letters were significantly different (*p* < 0.05). BHT = butyl hydroxyl toluene (positive control)

Antioxidant activity of the extracts was then assessed using two different methods, the FRAP and DPPH assays. The effect of selected extracts on FRAP and DPPH measured in this study are shown in Table 2 and Fig. 1, respectively. FRAP, a measure of antioxidant power, determines the reducing ability of an antioxidant reacting with a ferric-tripyridyltriazine (Fe^3+^-TPTZ) complex to form coloured ferrous-tripyridyltriazine (Fe^2+^TPTZ). Test samples that favour reduction of the complex from Fe^3+^-TPTZ to Fe^2+^-TPTZ, with indication of an intense blue colour development confirms the presence of a reductant ie., antioxidant [38]. FRAP values in the selected sample extracts ranged from 26.46 ± 1.73 to 453.30 ± 51.79 μg AAE/mg (Table 2). *P. banksii* leaf and fruit extracts and the *P. pubescens* leaf extract were significantly higher (*p* < 0.05) whereas the *M. pinnata* inner bark extract had the lowest FRAP values. The DPPH assay is a measure of the reduction of alcoholic DPPH solution in the presence of a hydrogen-donating antioxidant due to the formation of a non-radical form, DPPH-H. In this assay, the purple colour is reduced to a yellow coloured diphenylpicrylhydrazine by the test sample containing antioxidant [39]. For the tested extracts, all except *M. pinnata* inner bark extract showed very high radical scavenging activity (Fig. 1).

Several reports have shown that plant derived natural products are effective free radical or active oxygen scavengers which are attributed to high levels of phenolics and flavonoids [7, 40]. In this study, total phenolic and flavonoid content were comparable to previous studies reported on Australian food plants [41], but relatively higher than the other Australian medicinal plants [21]. In the present study, the total phenolic content of the medicinal plant extracts significantly correlated with FRAP (r ^2^ = 0.7568, *p* < 0.001) and DPPH (r ^2^ = 0.5684, *p* < 0.01), respectively (Table 3). However, there was no correlation observed between the total flavonoid content and FRAP or DPPH (Table 3). Differences in correlations for antioxidant activity and flavonoid and total phenolic content have been reported previously [42]. The result may suggest that components other than flavonoids are responsible for antioxidant effects.

**Table 3:** Pearson’s correlations between total phenolic, flavonoids contents and antioxidant activities of tested Australian medicinal plant extracts

Antioxidant activities	Pearson’s correlations (r^2^)	
	Total phenolic	Total flavonoids
FRAP ability	0.7568^b^	0.2932
DPPH radical scavenging ability	0.5684^a^	0.1765

a, b indicate significance at *p* < 0.01 and *p* < 0.001 respectively

*FRAP*, ferric reducing antioxidant potential, *DPPH* 1,1-diphenyl-2-picrylhydrazyl

### α-amylase and α-glucosidase inhibition

Extracts were screened to examine their inhibitory activity to enzymes relevant to the management of diabetes. A common therapeutic approach used in controlling non-insulin dependent hyperglycaemia is through inhibition of α-amylase and α-glucosidase that control the breakdown and absorption of glucose and its precursors in the small intestine. For α-amylase inhibition, IC_50_ values for the extracts ranged from 160.20 ± 27.92 to 310.17 ± 12.88 μg/mL (Table 4). The IC_50_ values for α-amylase inhibition of *P. banksii* (leaves, fruits and roots), *P. pubescens* (leaves and fruits) and *M. pinnata* (inner bark) were significantly lower (*p* < 0.05) in comparison to other sample extracts*.* For α-glucosidase inhibition, IC_50_ values ranged from 167.83 ± 23.82 to 350.23 ± 24.05 μg/mL (Table 4). The IC_50_ values of *P. banksii* (leaves, fruits), *P. pubescens* (leaves) and *M. pinnata* (inner bark) extracts were significantly lower (*p* < 0.05) than *P. banksii* (roots), *P. pubescens (*fruits), *M. pauciflorum* (leaves) and *Grewia mesomischa* (root bark).

**Table 4:** IC_50_ values of tested Australian medicinal plant extracts on fluorescent AGEs, α-amylase, α-glucosidase and ACE

Plant species	Fluorescent AGEs (IC_50_ μg/mL)	α-amylase (IC_50_ μg/mL)	α-glucosidase (IC_50_ μg/mL)	ACE (IC_50_ μg/mL)
*Petalostigma banksii*				
Leaves	56.05 ± 6.10^ab^	166.50 ± 5.50^a^	167.83 ± 23.82^a^	266.27 ± 6.91^a^
Fruits	47.72 ± 1.65^a^	170.81 ± 6.45^a^	190.90 ± 19.93^a^	452.47 ± 38.51^b^
Roots	34.49 ± 4.31^a^	174.57 ± 5.36^a^	345.27 ± 16.33^b^	523.87 ± 14.46^c^
*Petalostigma pubescens*				
Leaves	160.74 ± 3.86^c^	160.20 ± 27.92^a^	185.43 ± 14.05^a^	313.37 ± 29.33^a^
Fruits	83.52 ± 2.02^b^	187.90 ± 11.33^a^	348.13 ± 28.71^b^	597.53 ± 7.48^c^
*Memecylon pauciflorum*				
Leaves	76.66 ± 14.50^b^	270.47 ± 17.48^b^	350.23 ± 24.05^b^	609.47 ± 30.92^c^
*Millettia pinnata*				
Inner bark	71.48 ± 16.40^b^	188.63 ± 12.69^a^	183.83 ± 66.30^a^	695.17 ± 15.38^e^
*Grewia mesomischa*				
Root bark	50.51 ± 6.77^ab^	310.17 ± 12.88^c^	299.30 ± 9.10^b^	630.33 ± 11.77^d^

Values are expressed as mean ± SD, *n =* 3

Data in the same column marked with different letters were significantly different (*p* < 0.05)

A previous study on medicinal plant extracts, reported IC_50_ values for α-amylase ranging from 2070 to 13,740 μg/mL and α-glucosidase ranging from 2740 to 20,950 μg/mL [20]. These values are relatively high in comparison to the extracts tested in the present study and could be attributed to extraction techniques. The IC_50_ values of enzyme inhibition presented in our study were slightly higher compared to a previous report on Australian medicinal plant extracts [21], but were comparable to other medicinal plants [43, 44]. It has been reported that polyphenols, flavonoids and the antioxidant constituents of medicinal and food plants inhibit α-amylase and α-glucosidase [7, 43, 44]. In the present study, the IC_50_ values for both α-amylase and α-glucosidase were poorly correlated with the total phenolic, flavonoid, DPPH and FRAP (Table 5). The lack of correlation between the total phenolics and enzyme inhibitory activities has been previously reported [41, 45]. The various relationships reported in the literature clearly suggest that the total phenolics and their antioxidant capacities do not define hyperglycaemia related enzyme inhibition. This indicates the inhibitory actions of the tested extracts on α-amylase and α-glucosidase may be mediated by the action of constituents other than the phenolic and flavonoid compounds.

**Table 5:** Pearson’s correlations between the IC_50_ values for fluorescent AGEs, α-amylase, α-glucosidase and ACE versus total phenolic, flavonoids contents and antioxidant activities of tested Australian medicinal plant extracts

Assays (IC_50_)	Pearson’s correlations (r^2^)			
	Total phenolics	Total flavonoids	FRAP	DPPH
α-amylase inhibition	−0.3905	−0.1191	−0.2948	−0.1758
α-glucosidase inhibition	−0.3018	−0.1167	−0.3227	−0.3054
Fluorescent AGE inhibition	−0.1594	−0.3205	−0.2393	−0.3460
ACE inhibition	−0.7749^b^	−0.2460	−0.8451^b^	−0.5101^a^

a, b indicate significance at *p* < 0.05 and *p* < 0.001 respectively

*FRAP* ferric reducing antioxidant potential, *DPPH* 1,1-diphenyl-2-picrylhydrazyl, *AGE* advanced glycation endproducts, *ACE* Angiotensin converting enzyme

### Antiglycation

Extracts were assessed for inhibition of formation of advanced glycation endproducts (AGEs) in a model of protein glycation. AGEs are classified into two major groups: fluorescent (pentosidine, crosslines, and imidazolones) and non-fluorescent (*N* ^ε^-carboxymethyllysine and *N* ^ε^-carboxyethyllysine) [11, 12]. The formation of AGEs occurs through multiple processes related in part through ROS [9]. Medicinal plants have demonstrated their ability to inhibit the process of protein glycation, thus preventing alteration of the biological activity of protein, their degradation and conversion to AGEs [22, 23]. To our knowledge there are no reported studies on antiglycation activities of Australian medicinal plants. In the present study, an in vitro screening method was employed to determine the inhibitory effects of extracts on formation of fluorescent AGEs. The model system used a high concentration of reducing sugar to speed up the glycation process as glycation occurs very slowly under physiological conditions. Phosphate buffer (0.2 M, pH 7.4) increases the proportion of the open chain, reactive form of the reducing sugar in solution thus favouring the kinetics of protein glycation including formation of fluorescent AGEs [46]. A temperature-time at 37 ± 1 °C for 3 weeks was chosen to achieve minimally-modified protein under physiological conditions and to prevent any deterioration of the bioactive compounds in the plant extracts*.* Fluorescence intensity was determined using isolated protein with appropriate dilution to reduce quenching effects of plant extracts and coloured solutions due to glycation.

The antiglycation potential of the selected extracts were compared on the basis of their IC_50_ values and ranged from 34.49 ± 4.31 to 160.74 ± 3.86 μg/mL (Table 4). Of the selected samples, *P. banksii* fruits and roots had significantly lower (*p* < 0.05) levels, whereas *P. pubescens* leaves showed the highest (*p* < 0.05) antiglycation IC_50_ value. In *P. pubescens*, different plant components indicated different levels of antiglycation potential (Table 4). This suggests that the degree of antiglycation activities could vary from plant to plant and in different tissues from the same plant. It is suggested that the ability to reduce the formation of AGEs is closely related to the antioxidant properties of food and medicinal plants [22, 47]. Our studies showed no correlation between the antiglycation potential and the antioxidant activities (FRAP, DPPH) (Table 5). This indicates the efficiency of antiglycation potential by the tested extracts as an independent pathway rather than their ability in scavenging free radicals formed during protein glycation. Other mechanism of antiglycation such as blocking carbonyl or dicarbonyl groups in reducing sugars, breaking the cross-link structures in the formed AGEs and inhibiting the formation of late-stage Amadori products have been reported through use of natural products [47, 48]. Further comprehensive studies of the extracts tested in this study are required to evaluate the antiglycation mechanisms described above.

### ACE inhibitory activities

The plant extracts were also tested for ACE inhibitory activities. ACE is another enzyme that has an impact on metabolic syndrome - the stimulation of ACE causing high blood pressure. Inhibition of ACE by medicinal plant extracts to reduce hypertension has been previously reported [43, 44]. Recently effects of polyphenolic-rich fractions of the native Australian herbs and foods on ACE inhibition have been reported [41, 49], however, to our knowledge the present study is the first to report on the role of Australian medicinal plants on ACE inhibitory activities. For ACE inhibition, IC_50_ values ranged between 266.27 ± 6.91 to 695.17 ± 15.38 μg/mL (Table 3), with *P. banskii* leaves and *P. pubescens* leaves showing comparable and higher ACE inhibitory activities. *M. pinnata* inner bark extracts showed significantly (*p* < 0.05) lower ACE inhibitory activities. Results from our studies were comparable to ACE inhibitory activities of Lebanese traditional medicinal extracts reported previously [44]. Further, another study with different extracts of *Gynura divaricata* extracts has shown IC_50_ values for ACE inhibition ranging from 370–1540 μg/mL [43]. In the present study, a negative correlation between IC_50_ values of ACE inhibitory activities and total phenolics (r^2^ = −0.775, *p* < 0.001), FRAP (r^2^ = −0.845, *p* < 0.001) (Table 5) were seen in the tested extracts. In addition, moderate negative correlation between IC_50_ values of ACE inhibition and DPPH (r^2^ = −0.510, *p* < 0.05), but not the flavonoid content were found (Table 5). *P. banksii* leaves and *P. pubescens* leaves have shown lower IC_50_ values for ACE inhibition than the previous studies [43, 44], suggesting that a lower concentration of these extracts can be used to obtain 50 % inhibition of ACE. These results suggest that the Australian medicinal plants could be further investigated for treating hypertension through ACE inhibition.

### Previous research on the tested plant species

There have been limited previous studies of the medicinal activities of Australian species tested in this study. Extracts of different parts of *P. pubescens* and *P. banksii* were found to have promising antiglycation and enzyme inhibitory activities which have not been previously reported. Extracts of *Petalostigma s*pecies including *P. pubescens, P. augustifolium* and *P. triloculare* have previously been found to exhibit antimicrobial activity [50, 51], however very little is known of the chemistry of the genus. A GC-MS analysis of a fruit extract of *P. triloculare* showed the presence of low molecular weight compounds including acetic acid, 2,2-dimethoxybutane, decane, undecane, 1,2-benzenediol, 1,2,3-benzenetriol and benzoic acid [51]. The heartwood of *P. pubescens* has also been shown to contain diterpenoids including a cytotoxic diterpene (−)-sonderianol [52]. Another species, *P. sericea,* has been found to contain oleanolic acid [53]. No previous studies of this genus have examined effects on carbohydrate metabolism, antiglycation activities or ACE inhibition, and the possible active constituents are unknown.

Various species in the genus *Grewia* have uses in traditional medicine systems from a number of countries [54] and some species have been studied for their antioxidant effects. However there are no previous reports examining the antioxidant and enzyme inhibitory of effects of the Australian species *G. mesomischa* examined in this study.

The species *Memecylon pauciflorum* occurs naturally in Northern Australia and also occurs in SE Asia, southern Malesia and New Guinea [55]*.* Other *Memecylon* species used in traditional medicine in India including those reported as *Memecylon umbellatum*, *M. talbotianum, M. edule* and *M. malabaricum* have been studied for their medicinal effects including antioxidant [56, 57], anti-inflammatory [58, 59] and in vivo antidiabetic effects [60].

*Millettia pinnata* (synonym *Pongamia pinnata*) has a broad distribution from India, through central and south-eastern Asia, Indonesia and into northern Australia. *M. pinnata* grown in India has been studied for a number of activities. An extract of the leaves of the plant collected in Tamil Nadu, India was recently found to have moderate antioxidant activity and significant nitric oxide scavenging activity [61]. The total phenolic content of the leaf extract of *M. pinnata* was reported in that study as 4.1 ± 1.5 mg/g (μg/mg), lower than the total phenolic content of inner bark extract reported in our study (Table 2). Extracts of the stem bark have been shown to have antihyperglycaemic activity in diabetic mice and recently have been shown to decrease blood glucose levels, and improve electrocardiographic and hemodynamic parameters and improve oxidative stress in a model of cardiomyopathy in diabetic rats [62]. The stem bark extract has been shown to contain alkaloids, terpenoids (including triterpenes), steroids, flavonoids and volatile oils, with the isolated triterpenoid cycloart-23-ene-3β, 25-diol (B2) possessing antidiabetic activity and antioxidant activity in diabetic animals [63]. The bark has also been shown to have in vitro wound healing activity [64]. As *M. pinnata* has been noted to be a variable species and a number of varieties [65] it would of interest to further compare the medicinal activities and chemistry of *M. pinnata* native to northern Australia with that in other countries.

The plant extracts examined in this study, in particular the *Petalostigma* species tested, show potential for development as herbal or functional food products for the management or prevention of hyperglycaemia and its related complications. Further research is now needed to focus on isolation and characterization of bioactive compounds from these medicinal plants. This will be important for standardisation of the extracts and further understanding of the mechanisms underlying inhibition of protein glycation. While these extracts have long history of traditional use for inflammatory symptoms there is also a need to further evaluate their safety as part of any product development.

## Conclusion

This study reports for the first time on the potential of selected Australian medicinal plant extracts to inhibit protein glycation and enzymes related to hyperglycaemia and hypertension. The evidence produced in this study is useful to design further studies and investigate the antiglycation agents for the management of diabetic complications in vivo.

## Additional file

Additional file 1: Excel spreadsheet. Sheet 1. Sample codes. Sheet 2. IC_50_ calculations for anti-glycation, α-amylase, α-glucosidase and ACE inhibition. Sheet 3. Data for total phenolics, flavonoid content, FRAP and DPPH. (XLSX 19 kb)

## Abbreviations

AAE: Ascorbic acid equivalence
ACE: Angiotensin converting enzyme
AGEs: Advanced glycation endproducts
BHT: Butyl hydroxyl toluene
BSA: Bovine serum albumin
DPPH: 2, 2-diphenyl-1-picryl hydrazyl
FRAP: Ferric reducing anti-oxidant potential assay
GAE: Gallic acid equivalents
IC50: 50 % inhibitory concentration
QE: Quercetin equivalents
